# How to Tell If Animals Can Understand Death

**DOI:** 10.1007/s10670-019-00187-2

**Published:** 2019-12-13

**Authors:** Susana Monsó

**Affiliations:** Unit of Ethics and Human-Animal Studies, Messerli Research Institute, University of Veterinary Medicine Vienna, Vienna, Austria

## Abstract

It is generally assumed that humans are the only animals who can possess a concept of death. However, the ubiquity of death in nature and the evolutionary advantages that would come with an understanding of death provide two prima facie reasons for doubting this assumption. In this paper, my intention is not to defend that animals of this or that nonhuman species possess a concept of death, but rather to examine how we could go about empirically determining whether animals can have a concept of death. In order to answer this question, I begin by sketching an account of concept possession that favours intensional classification rather than mere extensional discrimination. Further, I argue that the concept of death should be construed as neither binary nor universal. I then present a proposal for a set of minimal conditions that must be met to have a concept of death. I argue that having a minimal understanding of death entails first expecting a dead individual to be alive, and then grasping its non-functionality and irreversibility. Lastly, I lay out the sort of observational and experimental evidence that we should look for to determine whether animals have the capacity for a minimal comprehension of death.

## Introduction

It is generally assumed that humans are the only animals who can possess a concept of death. The prevalence of this idea is exemplified by the debate on the ethics of killing nonhuman animals (hereafter ‘animals’). Within this debate, much of the discussion revolves around whether the fact that animals cannot understand death affects the extent to which death harms them. Authors such as Cigman (1981), Varner (2012, p. 147), Rollin (2015), and Belshaw (2015) argue that animals’ lack of a concept of death entails that (painlessly) killing them does not harm them, or harms them to a much lesser extent than it does humans. Other authors, like Regan (2004), DeGrazia (1996), Harman (2011), and Bradley (2015), consider that death can still be fully harmful in the absence of a concept of death. However, neither side of the debate questions the very idea that animals lack a concept of death. This premise is usually offered without any empirical justification by one side, and has remained largely unquestioned by the other. Cigman, for example, simply states: “It is only by an imaginative leap that possession of [the concept of death] seems attributable to animals” (Cigman 1981, p. 59). And Regan, who extensively criticises Cigman’s views, nevertheless agrees with her that it is “doubtful” that any animal can understand death (Regan 2004, p. 111).

Most philosophers who have discussed animals’ relation to death have taken for granted that only humans can grasp this notion.^1^ Upon reflection, however, one finds that there are at least two prima facie reasons for thinking that some animals may understand death. The first one is the fact that death is ubiquitous in nature (Allen and Hauser 1991, p. 228). Wild animals have very high mortality rates, due to factors such as disease, predation, lack of resources, natural disasters, and human-related causes. In addition, the vast majority of species follow a reproductive strategy that makes high numbers of animals come into existence only to die before reaching maturity (Horta 2015). Wild animals who live long enough will therefore encounter dead individuals sooner or later. This contrasts with many human lives. It might make sense for those of us who inhabit Western societies to construe death as an *abstract* concept (e.g. Rollin 2015, p. 44), since unless we have certain specific jobs or are extremely unlucky, we usually go through our lives having seldom encountered death. For animals in the wild (and for humans in certain societies), death is not at all abstract. It is something very tangible and very present. This ubiquity may allow those animals who have conceptual abilities to acquire a concept of death.

The second prima facie reason for questioning the assumption that animals lack a concept of death are the evolutionary advantages that would come with it (Allen and Hauser 1991, p. 228). First, a concept of death could help animals learn about new threats in the environment, by being able to quickly process that certain things are deadly. Further, since corpses are sources of disease, being able to recognise them and avoid them could have health benefits. In the case of social animals, having a concept of death could also allow individuals to grasp that when a group member dies this means that a competitor or a resource within the group no longer exists, which could be advantageous in certain situations. For scavengers, being good at recognising dead animals has clear survival value. And lastly, when it comes to the deaths of infants, being able to understand what happened could allow parents to better process that energy or resources no longer need to be invested in the deceased individual. Due to all of these advantages, it is plausible to think that natural selection may have favoured the development of a concept of death in some nonhuman species.

These two prima facie reasons are far from definitive. With respect to the ubiquity of death, one could counter-argue by saying that there are many phenomena that are ubiquitous in nature and of which animals likely have no concept, such as physical forces or chemical stimuli. With respect to the evolutionary advantages of a concept of death, it has been suggested that evolution may have in fact selected *against* it, since “the resulting overwhelming fear would be a dead-end evolutionary barrier, curbing activities and cognitive functions necessary for survival and reproductive fitness” (Varki 2009, p. 684). These objections, in turn, could be subject to counter-arguments.^2^ In this paper, however, I do not want to settle this issue from the armchair. My intention is not to defend that animals of this or that nonhuman species possess a concept of death, but rather to examine how we could go about *empirically determining whether animals can have a concept of death*. In order to answer this question, in the following section I will begin by sketching an account of concept possession that favours intensional classification rather than mere extensional discrimination. Further, I will argue that the concept of death should be construed as neither binary nor universal. I will then present, in Sect. 3, a proposal for a set of minimal conditions that must be met to have a concept of death. Lastly, in Sect. 4, I will lay out the sort of observational and experimental evidence that we should look for to determine whether animals have the capacity for a minimal comprehension of death.

## What Does It Mean to Possess a Concept?

In order to empirically determine whether animals can possess a concept of death, the first thing that needs to be clarified is what it means to possess a concept. Unfortunately, there is a huge amount of literature on the topic of concept possession and I cannot attempt to do justice to the different positions in this debate without this turning into a whole different paper.^3^ Therefore, to keep things simple, I will take as my starting point the account of concept possession that emerges from taking together three of the most influential papers on animal concepts: Allen (1999), Glock (2000), and Newen and Bartels (2007). In the account of concept possession that I will sketch as a result, two fundamental ideas will emerge: concept possession is a classificatory skill, rather than a merely discriminatory one, and the concept of death is neither binary nor universal.

### Concept Possession as Intensional Classification

There are small differences in the views defended by Allen (1999), Glock (2000), and Newen and Bartels (2007), but there is also a substantial amount of common ground. The first point of consensus is that *mere discrimination is not enough* for concept possession (Allen 1999, pp. 36ff; Glock 2000, pp. 45ff.; Newen and Bartels 2007, p. 288). An example that is often cited to make this point is especially handy because it refers, precisely, to the concept of death: this is the example of necrophoresis in ants (Allen and Hauser 1991, p. 231; Allen 1999, p. 36; Newen and Bartels 2007, p. 287). As is well known, ants will systematically remove dead conspecifics from their nests, something that presupposes the capacity to *discriminate* dead individuals. However, we are quite certain that necrophoresis is not mediated by the use of concepts, because it is very closely tied to the presence of certain perceptual cues in the environment. Dead ants give off oleic acid, and this is a chemical that triggers necrophoresis.^4^ We know from experiments performed on ants that they will remove from the nest any object that is sprayed with oleic acid, including live ants (Wilson et al. 1958). In this last case, the detection of oleic acid triggers necrophoresis and the ants are not able to make the inference from the fact that the being they are carrying is moving to the conclusion that she is not dead. This shows us that they are likely not operating with any concept of death. Ants can recognise dead individuals, but not recognise them *as* dead (Allen and Hauser 1991, p. 231). The same appears to be the case with rats. In this case, the olfactory detection of putrescine or cadaverine, which are given off by decaying rat carcasses, triggers burying behaviour. No conceptual mediation is apparently present, since rats have been found to bury anaesthetised conspecifics and pieces of wood that have been sprinkled with either of these two components (Pinel et al. 1981).

Discrimination, then, should be distinguished from what Glock terms *classification* (Glock 2000, pp. 45ff.), which, in contrast to the former, is a conceptual ability. The mere ability to discriminate dead individuals, which is what ants and rats seem to possess, is based solely on perceptual cues and triggers a reaction that cannot be inhibited or modified, but is instead rigid. At the same time, discrimination lacks the normative dimension that would allow us to say that when an ant removes a conspecific that has been sprayed with oleic acid she is making a mistake in a rule that she was trying to follow, for she could not have acted in any other way (Ibid., p. 46). The case of conceptual classification is different. Here we find relative independence from perceptual cues and the possibility of behavioural flexibility, so that different perceptual cues can trigger the same behaviour, and different behaviours can be triggered by the same cue (Allen and Hauser 1991, p. 231; Allen 1999, p. 36; Glock 2000, p. 47; Newen and Bartels 2007, pp. 286–287). In addition, the animal may also choose to ignore the perceptual cue (Glock 2000, p. 47). Classification further incorporates a normative dimension that allows us to speak of mistakes in the application of a rule—when, for instance, the animal classifies as dead a being who is not actually dead—since the animal can now in principle realise that she has violated the rule she was intentionally trying to follow, i.e. realise that the being she had taken for dead is actually alive (Glock 2000, p. 46; Allen 1999). Classification also allows, at least in principle, the possibility of learning [for instance, learning to better distinguish dead from non-dead individuals after a mistake has been made (Allen 1999)].

The most important difference between classification and discrimination, for present purposes, is that discrimination is nothing but an *extensional* competence that allows an animal to distinguish things that possess a certain property, whereas classification entails grasping the *intensional* features of that property (Allen and Hauser 1991, p. 227; Newen and Bartels 2007, p. 297). So, for an animal to possess a conceptual ability to classify others as dead it is not so important that the animal be reliable in her ability to discriminate dead individuals. Instead, what is necessary is for the animal to have some grasp of *what it means* to be dead. Thus, in order to determine how to tell whether an animal possesses a concept of death we need to first establish what are the intensional features of death that the animal needs to have a grasp of.^5^ This task that will be undertaken in Sect. 3, but first we need to clarify some further notions about concept possession that are especially important in the case of the concept of death.

### The Concept of Death is Neither Binary nor Universal

Contrary to what is often assumed by animal ethicists, the concept of death should not be viewed in binary terms. Possessing a concept of death is not an all-or-nothing matter but rather something that is subject to gradation. This becomes clear once we consider the case of human children, who do not acquire a concept of death overnight. In fact, the scientific consensus is that it takes them an average of 10 years to fully master the concept of death (Kenyon 2001), but we credit them with *some* understanding of death before they reach this stage. If we accept a gradation in the case of human children, we should also accept it in the case of animals. However, when animal ethicists describe the concept of death in terms as demanding as the understanding of “the possibility of the impossibility of one’s being” (Rollin 2015, p. 52; following Heidegger, [1927]^1996^), they are using as benchmark the (educated) adult human concept of death. This sets the stage in a biased way, and it is also to a certain extent arbitrary. This is because human adults’ concept of death is also limited. We do not experience death directly while we are alive and can only speculate as to what it feels like or what happens after it. Even if we are convinced that the experience of death is simply the annihilation of one’s consciousness, it is beyond our imaginative powers to fully grasp this idea. So, if we were to favour a strict all-or-nothing approach to the concept of death, it might be more rigorous to conclude that adult humans lack a concept of death too. I take it that this would be an absurd conclusion, and that it is preferable to favour a graded account of the concept of death, where adult humans can be said to comprehend death to a very high, if perhaps not full, extent. Children at different developmental stages will, in turn, understand death to varying degrees. Within such a framework, the impartial way to phrase the question with respect to animals is not whether they can possess adult humans’ concept of death, but whether they can possess anything that counts as an understanding of death at all.

Animal ethicists not only speak as though the concept of death were binary, they also often assume that it is universal, that there is only one concept of death, *the* concept of death. In contrast, within debates on concept possession it is widely acknowledged that concepts are not universal, but that they vary across cultures, across individuals, and also across time for every one of us (Allen 1999, p. 35; Routley 1981, p. 290). Thus, it is only logical to suppose that the concept of death (if it indeed exists beyond the boundaries of our species) will vary across species. Different species may have different notions of death, but also different individuals within each species may have a different understanding of death, and within the lifetime of a single individual the concept of death she possesses may vary. Any empirical examination of whether animals understand death must take this variability into account.

## The Minimal Concept of Death

In this section I will develop a set of conditions that an animal must fulfil in order to be credited with a minimal understanding of death. Having granted that the concept of death is not universal, one could wonder why I opt for offering a set of minimal conditions, rather than constructing the concept of death as something more open-ended, such as a cluster concept. One of the reasons for this is that, while I acknowledge the need to incorporate variability, I also want to construct a concept of death that can serve to address the assumption that animals lack it. If we were to depart from an open-ended cluster concept, it is difficult to see how we could address this assumption. But more importantly, I believe that, while there are some variations, there are also commonalities that are present across all human contexts where it is plausible to speak of the existence of a concept of death. As I will argue, a concept of death that did not meet these necessary and sufficient conditions would simply be too alien for us to plausibly construe it as such, regardless of whether it was possessed by a human or an animal.

### The Seven Sub-components of the Scientific Concept of Death

In order to determine the minimal conditions that a concept of death must fulfil, it is useful to start from the set of sub-components of the concept of death that developmental psychologists use to determine the extent to which human children understand death. These sub-components correspond to the scientific understanding of death. Following Slaughter (2005), we can identify seven sub-components that make up the full-blown scientific concept of death:

1. *Non*-*functionality*: death implies the cessation of all bodily and mental functions.
2. *Irreversibility*: dead individuals cannot come back to life.
3. *Universality*: all living things, and only living things, die.
4. *Personal mortality*: death will also apply to oneself.
5. *Inevitability*: eventually, all living things must die.
6. *Causality*: death occurs due to a breakdown in the bodily functions.
7. *Unpredictability*: it is impossible to know in advance the exact timing of death.

In conceptions of death that don’t qualify as scientific, because they belong to a worldview that incorporates supernatural entities, some of these seven sub-components might not be present or be just partially present. For instance, some magical or religious worldviews might entail that some living things don’t die, which would mean a relinquishment of the sub-component *universality*. Or they might entail that only the bodily, but not the mental functions, are terminated with death, which would mean only a partial presence of the sub-component *non*-*functionality*. I’m committed to the view that there is a set of commonalities that allows us to speak meaningfully of a concept of death across different human contexts, and it is these commonalities that need to be captured in a minimal concept of death that can be used to study the case of animals. In order to identify these common elements, I will now examine the seven sub-components of the scientific concept one by one to see which of them are crucial and to what extent they need to be present in a minimal concept of death.

The first sub-component is *non*-*functionality*, the idea that death implies the cessation of all bodily and mental functions. As already mentioned, under some non-scientific conceptions of death, some of these functions may survive death. It would, however, seem outlandish to say that a Christian who believes her mental functions will continue existing after death lacks a concept of death or has one that is radically different from the scientific concept of death. A Christian and an atheist can still meaningfully communicate with each other about death, as is common in societies where secular and religious people co-exist. At the same time, someone who believed that *all* bodily and mental functions survive death would appear to both a Christian and an atheist as fundamentally confused or mistaken about what death is. Therefore, *some* non-functionality must be present in a minimal concept of death. The functions that are understood to terminate with death will, in turn, be those that are conceived as *characteristic* of live individuals. Under the Christian conception, these will be only the bodily functions, as mental functions are thought to be characteristic of both live and dead individuals. So, a minimal concept of death must incorporate non-functionality, while leaving room for variations in the cluster of functions conceived as characteristic of living beings. This variability is also relevant for the case of animals. We can’t expect an animal to understand that dead individuals lack functions that the animal doesn’t know that living beings have. So, for instance, we can’t expect an animal who doesn’t have a notion of digestion to understand that death implies the cessation of digestion. If an animal can understand non-functionality at all, this understanding is going to mirror her understanding of functionality. Therefore, we can expect an animal who has a minimal concept of death to understand that dead beings don’t do the things *that she understands that living beings characteristically do*—things like moving, eating, mating, grooming, playing, etc.

The second sub-component, *irreversibility*, is the idea that dead individuals cannot come back to life. Very young children, when they first learn about death, tend to view it as something that is reversible, such as falling ill or going to sleep (Speece and Brent 1984, p. 1673). A definition of the minimal concept of death should be able to capture why we believe this to be a fundamental confusion. At the same time, however, we want the minimal concept of death to encompass conceptions of death with some element of reversibility, such as those that involve reincarnation. I suggest that the notion of irreversibility should be present in the minimal definition, but attached to the cluster of functions that are thought to terminate with death. Thus, an individual who believes in reincarnation will still have a concept of death, because the reincarnation is thought to occur *in another body*, so there is no reversion of the (bodily) functions that ended with death. This definition allows us to capture why an adult who believed death to be completely reversible would be confused or would have a concept of death that is so radically different from ours as to warrant us not classifying it as such. At the same time, it allows us to say that an animal who understands that the dead conspecific is not doing anything right now, but that she will get up and start moving at any moment, has not grasped the meaning of being dead, and is instead operating with a concept that comes closer to that of being asleep.

The following three sub-components, *universality*, *personal mortality*, and *inevitability*, can be taken as a set, because if *universality* is true, that is, if all living beings and only living beings die, then personal mortality and the inevitability of death are entailed. These three sub-components are *not* required for a minimal understanding of death. This is because it is in principle possible for an individual to understand death as something that happens only to *some* individuals, and still possess an understanding of death. That this understanding is not precluded by a lack of these three sub-components is illustrated by the following thought experiment. Imagine that we were to learn that not all of us are going to die, or that we ourselves are not going to die, or that there is something we can do to postpone death forever. This would not fundamentally change what it means to die, although it would undoubtedly change how we experience life. In such a context, if we were to hear that Smith died, it would still mean the same thing: that Smith has lost the cluster of functions characteristic of living beings and will stay in that state forever. Although in such a world there would arguably be an extra dimension of tragedy to Smith’s death, we can still make sense of it with our regular understanding of death. So, these three sub-components are not necessary for a minimal comprehension of death.

The following sub-component, *causality*, would certainly be advantageous to an animal who possessed it. Being able to understand the causality behind death could increase her fitness by allowing her to better avoid potential sources of danger. However, a minimal concept of death does not require an understanding of the causality behind death, for the simple reason that understanding what causes *X* in general is not necessary for having a concept of *X*. Thus, I can have a concept of arthritis without knowing what causes arthritis. Becoming familiar with the causality behind arthritis simply makes my concept more complex and perhaps more useful, but the concept of arthritis does not require it. Therefore, we should also relinquish the sub-component *causality*, despite the evolutionary advantages that may come from grasping it.

With respect to the final sub-component, *unpredictability*, the first thing that needs to be said is that it is not true that death is inherently unpredictable. In certain contexts, such as the execution of someone with a guillotine, the timing of death can be predicted with a high level of precision. But more importantly, if hard determinism were true and we were able to calculate the exact timing of all of our deaths, this would, again, not fundamentally change what it means to die. It would probably entail a radical shift in the way we live our lives, but dying would still mean an irreversible cessation of those functions that are characteristic of living beings. Therefore, unpredictability is not required for a minimal understanding of death, either.

### A Proposal for a Minimal Concept of Death

The previous sub-section allowed us to conclude that the crucial sub-components of the concept of death are *non*-*functionality* and *irreversibility*. With this in mind, here is my proposal for what a minimal concept of death looks like:

A creature can be credited with a minimal concept of death once she classifies some dead individuals as dead with some reliability, where ‘dead’ is understood as a property that pertains to beings who:

(a) are expected to have the cluster of functions characteristic of living beings, but

(b) lack the cluster of functions characteristic of living beings, and

(c) cannot recover the cluster of functions characteristic of living beings.

Several things need to be highlighted about this definition. First, a minimal concept of death presupposes a minimal concept of life. This means that this definition satisfies Newen and Bartels’ requirement for the presence of some contrastive concepts, which they postulate because they believe that concepts can only emerge “in a minimal semantic net by classifying the objects in contrastive classes” (Newen and Bartels 2007, p. 298). It seems indeed impossible to have a concept of death without some understanding of life. If one lacked a concept of life, inanimate entities would be indistinguishable from dead ones. So, for an animal to understand that another being is dead, the animal first needs to classify this individual as one who would typically be alive. This classification need not be understood in cognitively demanding terms. It can take the form of an implicit expectation that this being will do the sorts of things that beings of its kind usually do, such as vocalise in a certain way, move in a certain way, eat certain things, etc.

Further, possession of a minimal concept of death entails classification, rather than mere discriminatory capacities, so the emphasis is not placed on the animal’s capacity to recognise dead beings, but on her capacity to recognise them *as* dead. Thus, full reliability in the discrimination of dead individuals is not required. An animal can sometimes mistake beings that are asleep or in a coma for dead beings and still possess a concept of death. However, if the animal were to get it wrong *every single time*, then it would not make sense to credit her with an understanding of death, even a minimal one. So, *some* degree of reliability is required. That this reliability need only be partial is also implied by the absence of the sub-component *universality* in the minimal concept of death. The animal who has only a minimal understanding of death does not know that *all* living beings die. Thus, she may be capable of recognising as dead only dead conspecifics, and not members of other species. This does not preclude her from having a concept of death. As Allen and Hauser note, it is possible for an animal to have a concept of death but “not recognize that or… not care whether certain objects are capable of falling into its extension” (Allen and Hauser 1991, p. 237).

It is also important to highlight that this definition leaves room for variation across species, across individuals, and across time. This is the reason behind the vagueness of the phrase ‘the cluster of functions characteristic of living beings.’ Rather than listing the specific functions that an animal must know that dead individuals lack, this criterion is left open-ended to allow for variation. Just as in the case of humans pertaining to different cultural groups, different species may have a different understanding of the sorts of functions that characterise live individuals and that are terminated with death. For instance, for species characterised by high competitiveness, an important function of live conspecifics may be that they compete for mates or resources, while species that are more cooperative may think of live conspecifics prominently as resources within the group. This criterion also leaves room for variation across individuals, so that, for instance, individuals with very playful personalities may place special emphasis on the fact that dead individuals do not play. And there is also room for variation across time, since the cluster of properties attributed to live individuals, or the number of objects that are understood to fall under the extension of the concept of death, may vary as an animal matures or gains more knowledge. In addition, the definition incorporates this temporal dimension, presupposing that no animal will be born with a concept of death, but that this is something that can be acquired (if at all) as a result of an accumulation of experiences. The definition then provides us with criteria to (roughly) determine the point in time at which an animal can be credited with a minimal understanding of death.

## How Can We Tell If an Animal can Understand Death?

Having analysed the necessary and sufficient conditions for a concept of death, I now turn to considering how we could empirically determine if an animal possesses it. There are two main ways of studying the cognitive capacities of animals. One is the observational method, characteristic of ethology, which consists of the systematic observation of (wild) animal populations over extended periods of time in order to gather and interpret data on their behaviour and its underlying mechanisms. The second method is the experimental one, characteristic of comparative psychology, which consists of testing (captive) animals’ behavioural/emotional/physiological reactions under different conditions. The experimental method is often considered more reliable, since it can control for confounding factors and thus give a more precise characterisation of the mechanisms underlying animal behaviour. However, what is gained in reliability is often lost in ecological validity, since the situations the animals are placed in are often highly artificial and may thus be prone to false negatives. Therefore, any attempt to study the concept of death in animals should ideally combine observational and experimental studies. In this section, I will detail, firstly, the sort of evidence that ethologists should be on the lookout for and, secondly, the type of experiments that comparative psychologists could use to test for animals’ capacity to understand death.

### In Search of Observational Evidence of a Concept of Death in Animals

As noted, studies that are purely observational have the disadvantage of not being able to easily control for confounding factors that may be influencing animals’ behaviour. However, they come with two advantages that are especially valuable for a study of animals’ understanding of death. The first advantage is that these studies usually consist of the observation of animals in their natural habitat. Natural habitats entail more opportunities for animals to encounter dead individuals, since in zoos and other captive settings dying individuals are often removed from the group. Second, and relatedly, observational studies allow us to gather data on animals’ reactions to death without engaging in ethically questionable practices. As we will later see, in the case of experimental studies this is not at all easy.

In what follows, I specify the sorts of behaviours that could be evidence of either an understanding of death or a capacity to learn about death. Needless to say, a single anecdotal observation of any these behaviours can never be considered evidence of a concept of death. However, if many or most of the different behaviours illustrated here were consistently observed in a single species or individual, this would amount to enough converging evidence to support the thesis that animals of that species can understand death. The sorts of behaviour that scientists should then be on the lookout for are:

1. *Varied behaviour towards corpses*: Animals operating with a concept of death will be *classifying* dead individuals as such, as opposed to merely discriminating them. As discussed before, mere discrimination occurs when members of a species respond in very rigid, stereotyped ways to dead individuals, where these responses are mediated solely by perceptual cues. With mere discrimination (i.e. lack of conceptual abilities) we would then expect members of a species to exhibit very little variation in their reactions to one and the same perceptual stimulus. So, researchers should look for evidence of members of a species reacting in a variety of ways to the same perceptual stimulus (in this case, a dead individual). The exhibition of different sorts of behaviours (aggressive, exploratory, sexual) towards a carcass on behalf of a single individual also gives evidence of an uncertainty regarding how to respond the corpse (Piel and Stewart 2015, p. 18). This uncertainty could be a sign that the animal was expecting the dead individual to exhibit the sorts of functions characteristic of live individuals of its kind. Only when there is this expectation, and the animal can process its violation, can the animal start to learn about death.
2. *Unhygienic/maladaptive behaviour towards corpses*: As pointed out by Piel and Stewart (2015, p. 18), species who are hardwired to respond in stereotyped ways to carcasses usually exhibit hygienic responses to them, since these have high survival value due to the health hazard posed by decaying corpses. Hygienic behaviours include the removal of dead conspecifics from the nest exhibited by ants and the burying of corpses displayed by rats. As noted before, no conceptual mediation is needed in these cases and it is likely that these animals cannot develop a concept of death. The insistent manipulation of corpses seen in other animals (for reviews, see: Fashing and Nguyen 2011; Anderson 2016) could be considered maladaptive behaviour (Piel and Stewart 2015, p. 17), so it is unlikely to be hardwired and thus a potential sign of a capacity to learn about death.
3. *Different treatment of corpses vs. asleep individuals*: Asleep individuals exhibit a degree of non-functionality, but they differ from dead individuals in that they still show some signs of life (body heat, breathing…) and in that they typically wake up after some time. As argued before, animals who cannot grasp the difference between ‘dead’ and ‘asleep’ cannot be credited with a concept of death. Therefore, it is important to monitor whether animals show a different treatment of corpses versus asleep individuals, which would be a sign of different concepts underlying the interactions.
4. *Investigative behaviour towards corpses*: A close inspection of corpses—sniffing them, touching them, observing them, nudging them—has been witnessed in a variety of social mammals, including chimpanzees (Hosaka et al. 2000; Anderson et al. 2010; Stewart et al. 2012; van Leeuwen et al. 2016), monkeys (Bezerra et al. 2014; Yang et al. 2016), elephants (Douglas-Hamilton et al. 2006; McComb et al. 2006; Merte et al. 2009), and peccaries (de Kort et al. 2018). This sort of behaviour could allow animals to learn about the absence of functions that indicate life, such as movement or body heat, thus potentially allowing for the development of a concept of death.
5. *Aggressive behaviour towards corpses*: Behaving aggressively towards a carcass is also a way in which the animals can learn about the non-functionality of a corpse. Chimpanzees, for instance, have been witnessed shaking dead bodies, hitting them, or dragging them across the ground (Anderson et al. 2010; Stewart et al. 2012; van Leeuwen et al. 2016). This could possibly mean that the chimpanzees have not processed that the individual is dead, but scientists have speculated that these behaviours could be attempts to elicit a response from the dead conspecific, possibly as a way to determine whether or not she is actually dead (Anderson 2011, p. 411; van Leeuwen et al. 2016, p. 8). In addition, chimpanzees often display in front of corpses (Anderson et al. 2010; Stewart et al. 2012; van Leeuwen et al. 2016), a show of dominance that would elicit an immediate behavioural response on behalf of the receptor, if they were to be alive, thus giving the displaying chimpanzee a chance to learn about the non-functionality of corpses. Though aggressive behaviour towards corpses could therefore count as an investigation of their non-functionality, I list it as a separate criterion from investigative behaviour because aggression could also be an expression of frustration at the lack of response, and thus possibly a sign that the animal has begun to process non-functionality (Stewart et al. 2012, p. 4).
6. *Caring behaviour towards beings with limited functionality*: Evidence of animals’ grasp of the functions of live individuals could come from cases of ‘compassionate care-giving’ directed at dying individuals, such as has been witnessed occasionally in dolphins (e.g. Park et al. 2012) or elephants (e.g. Douglas-Hamilton et al. 2006). In the cited cases, the dolphins and elephants were apparently helping the dying individual stay afloat or stand up, respectively. Given that neither dolphins nor elephants usually spend their time helping fully functioning individuals swim or stand, these reports are evidence that in these cases they understood the functional limitations of these particular individuals. Another interesting place to look for evidence that animals might grasp non-functionality is to observe their reactions to disabled individuals. Disabled individuals are characterised by differing in some of the functions that typically characterise live individuals of their kind, so if animals tailor their behaviour to the limitations of disabled conspecifics [which sometimes happens (e.g. Turner et al. 2005; Matsumoto et al. 2016)], then it is an indication that they have a grasp of the range of functions that live conspecifics tend to share, a necessary condition for the acquisition of a concept of death.
7. *Mourning behaviour towards corpses*: The death of an animal could be stressful for members of her social group, for instance, if they shared a close relationship with her or if she was a valuable resource within the group. If animals respond with distress, sadness, or apathy to the death of a conspecific, this could be an indication of an understanding of the irreversibility of their current state. It is of course very difficult to determine whether a dead conspecific is the intentional object of an animal’s emotion, but in cases where the emotional displays are accompanied by repeated returning to or manipulation of the carcass, this gives us some suggestive evidence of their intentional object. Along these lines, an interesting behaviour to monitor is that of mothers insistently carrying the carcass of their offspring. This behaviour has been found in chimpanzees (Biro et al. 2010), gorillas (Warren and Williamson 2004), Barbary macaques (Campbell et al. 2016), gelada monkeys (Fashing et al. 2011), Japanese macaques (Sugiyama et al. 2009), dingoes (Appleby et al. 2013), sea otters (Kenyon 1969), seals (Rosenfeld 1983), and a variety of cetaceans (Reggente et al. 2016). The transportation of a dead infant may well be a sign that these mothers do *not* understand that their infant is dead. However, this interpretation could be questioned in at least those cases in which the mothers carry the carcass how they would not carry a live infant [e.g. monkeys have been witnessed carrying their dead offspring with one hand or in the mouth (Fashing et al. 2011)], or treating the corpse in ways that would be dangerous to a live infant [such as leaving it on the ground while they forage nearby, or allowing its head to be submerged while they drink (Anderson 2011, p. 411)]. Such careless treatment of corpses is a prima facie indication of an understanding of the differences in functionality between a dead and a live infant.
8. *Eventual ignoring or abandoning of a corpse*: Just as mourning behaviour can be an expression of an understanding of the irreversibility of death, so can the eventual ignoring or abandoning of a corpse. Acquiring a minimal concept of death means learning that the state of the dead individual is a permanent one, which should come with a realisation that they are not worth interacting with anymore. It is therefore of relevance to monitor when and how the animals decide to abandon or ignore a corpse, in search for instances where this could be concept-mediated. For instance, mothers who carry their dead offspring eventually let go of them. In some cases, this occurs before there is a hormonal change and, in others, long after menstrual cycles resume (Fashing et al. 2011, p. 406; Anderson 2016, p. R155). So, the abandonment of the corpse may not always be explicable in hormonal terms, and this could be evidence of some cognitive mediation, some explicit realisation that the infant is not functioning *any more*. The same goes for those cases in which animals are seen insistently returning to a carcass. In these cases, the animals will eventually abandon the corpse. If there are no other environmental factors that could explain it, the abandonment could again be the sign of a realisation that the state is a permanent one.
9. *Age or experience*-*relative difference in behaviour towards corpses*: As argued in Sect. 3, no animal will be born with a concept of death, and this is instead something that animals will acquire, if at all, as a result of their personal experiences. Thus, one final source of evidence would consist of behavioural differences across time or individuals depending on previous encounters with death. Observational studies often monitor the same group of animals for an extended period of time. This could give us the chance to observe whether there are any differences in the way a particular individual reacts to death as she gains more experience with it, or whether there are differences in how older or more experienced individuals react to death, as opposed to how younger or naïve individuals react to it. For instance, it is to be expected that older individuals or mothers who have lost several offspring will be quicker in abandoning a corpse, if indeed they have acquired a minimal understanding of death.

### How to Test for Animals’ Concept of Death

In the previous section, I illustrated the sorts of behaviours that ethologists should look for if they want to gather evidence of animals’ understanding of death. Although evidence from field studies has high ecological validity, it is unfortunately very difficult to interpret due to its anecdotal nature and the lack of controlled conditions. Therefore, observational evidence should be complemented with experimental studies, which allow us to tailor the situation in order to test for specific abilities in animals. In this section I will give some hints as to how we can use experiments to garner evidence of a concept of death in animals.

Animals’ understanding of death has rarely been experimentally tested, and in those cases in which it has, the experiments have mostly consisted of presenting the tests subjects with carcasses and observing their reactions. Kaplan (1973), for instance, presented mother squirrel monkeys with the dead bodies of their own and other infants. He found that the mothers whose offspring had died older were more likely to respond emotionally only towards the dead body of their own infant. McComb et al. (2006) presented African elephants with the bones of related and unrelated conspecifics, as well as heterospecifics. They found that these animals were equally interested in the bones of elephant kin and non-kin, and much less interested in the bones pertaining to other species. Swift and Marzluff (2015) found that corvids will avoid feeding in an area where a dead conspecific has been found and will display aggressive, anti-predator behaviour directed at humans who have been seen holding a dead conspecific. While these studies are interesting, on their own they tell us little more than what can be gathered solely from observational studies.

Allen and Hauser (1991) presented two theoretical designs to test for animals’ concept of death. The first one would involve a playback experiment with vervet monkeys, where monkey mothers whose offspring had recently died would be played distress calls of their infant from a concealed speaker. Given that vervet monkeys are very good at distinguishing calls from specific individuals, such an experiment could give us insight into the mothers’ capacity to process that their own infant died. However, given how attached monkey mothers appear to be towards their offspring, subjecting them to such a form of deception seems unethical, to say the least (an analogous experiment performed on humans would be unthinkable). Allen and Hauser also note the difficulties involved in interpreting the mothers’ possible reactions to such playback calls, as well as the near impossibility of adding proper experimental controls that meet ethical standards (Ibid., pp. 233ff.).

The second experiment that Allen and Hauser propose would consist of administering a drug that causes unconsciousness and depression of vital signals to a member of a social group of animals. The effects of this drug would eventually wear off, and then the experimenters would administer the drug to the same animal a second time, to see if the others would anticipate that she would eventually wake up. In a third condition, the drug would be administered to a different individual, to see if the others could generalise what they had learned to further cases (Ibid., p. 236). While this experiment could be considered more ethical, it is doubtful that it can provide us with evidence regarding animals’ understanding of death. This is because, even if the drug causes a depression in the vital signals, no drug can fully imitate the non-functionality of a dead body (its coldness, absolute stillness, or rigor mortis). In addition, given that the drug has only temporary effects, it does not allow us to test for animals’ understanding of irreversibility. Allen and Hauser propose to compare the animals’ reactions to the drugged individuals with those displayed towards dead ones (Ibid.), but it is difficult to see how this can give us better evidence than a comparison of animals’ reactions to asleep versus dead conspecifics.

If we want to obtain evidence of animals’ capacity to understand death, while at the same time maintaining ethical standards, we need to approach this in a completely different way. My proposal is to test the animals with the use of artefacts. The idea would be to develop a mechanism of some sort that the animal has to utilise in a certain way in order to obtain a reward. In the test condition, the mechanism would break or shut down; it would lose its functionality. Moreover, it would do so in a way that would be irreversible, and this irreversibility would be clearly visible to anyone who understood the functions of the mechanism. At this point, if the animal wants to access the reward, she has to realise that she cannot do so any longer with this particular artefact, and must either make use of another one or find some other alternative. Needless to say, this is hardly a concrete proposal and the details would need to be much more thoroughly developed. In addition, it would need to be designed in a way that takes into account the species-specific physiology and behaviour. It might be some sort of tool if we’re testing apes or corvids, or a touch screen if we’re testing dogs. The details are unimportant for present purposes. What matters is that the test should be designed in a way that the animal could not pass it without an ability to process non-functionality and irreversibility.

Does this give us *direct* evidence of the animal’s understanding of death? Clearly, it doesn’t, given that the animal is not actually interacting with a dead individual. However, if an animal passes the test, it shows us that she has the *necessary cognitive requirements* to understand death. It shows us that she can *process the crucial sub*-*components* of a minimal concept of death. Thus, it gives *indirect* evidence of her capacity to understand death. Coupled with observational reports of the behaviours detailed above in the same species, this would amount to enough evidence to question the common assumption that animals cannot acquire a concept of death.

One final objection can be anticipated at this point: if the broken artefact possesses both non-functionality and irreversibility, does this mean that it counts as dead? Would that not invalidate the minimal concept of death I have defended? It would not, because to determine that something is dead, it is not enough to say that it is irreversibly non-functional. It is also crucial to *first classify it as something that would typically be alive*. Since artefacts typically don’t possess life, they cannot die either. In fact, it is possible to turn this objection on its head and argue that to get something closer to *direct* evidence of an animal’s concept of death we could actually design the artefact in a way that emulates life, by making it, for instance, move in a certain way, emit certain odours, or make certain noises. Although the artefact would not *actually* be alive, an animal could misclassify it as such and thus later pass the test by misclassifying it as dead. The fact that the test would be passed on the basis of a misclassification would not invalidate the evidence, given that, as argued, full reliability is not necessary for possession of a concept of death. While designing the artefact this way might yield even more relevant evidence, I am wary of suggesting it as an approach, since the temptation might then be to make the artefact as close as possible in feel and appearance to the test subjects’ conspecifics, and thus easily develop into an ethically problematic experiment.

## Conclusion

In this paper, I have not attempted to answer the question of whether animals of any particular species can possess a concept of death. Although I have cited some suggestive evidence, my aim was to offer a set of guidelines for those researchers who want to investigate whether animals can possess a concept of death. I have argued that having a minimal understanding of death entails first expecting a dead individual to be alive, and then grasping its non-functionality and irreversibility. I have given a list of nine behaviours that ethologists should look for to gather observational evidence of an understanding of death, and provided a rough sketch of how this can be complemented with experimental evidence. If many or most of the behaviours listed are observed in a species that can also pass a suitably-designed experiment, then we could invalidate the assumption, prevalent amongst animal ethicists, that no nonhuman species can acquire a concept of death.

As a final objection, one could point out that the minimal concept of death I have presented does not include the sub-component *personal mortality*. It could be argued that when animal ethicists assume that animals lack a concept of death they are focusing on this subcomponent. That is, they are discussing whether death harms an individual when that individual is not aware of *her own* mortality. Therefore, it could be argued, animal ethicists can continue to assume that animals lack a concept of (their own) death. However, while the minimal concept of death I have presented does not include the sub-component *personal mortality*, a minimal concept of death need not be the end point for an animal who is learning about death. The concept of death is something that evolves and acquires more complexity over time. It is, therefore, in principle possible for animals who have lived long enough to witness many deaths of conspecifics, to eventually reach, through inductive means, the conclusion that they themselves will also die. This seems unlikely, but not impossible. And even if it is beyond animals’ cognitive prowess to ever grasp the *inevitability* of their own death, it seems plausible that an animal who has witnessed and processed that several others have died due to a certain cause will reach the conclusion, when faced with that very threat, that her own life is at risk. Once we knew that an animal has a minimal concept of death, these possibilities would be in principle open, and thus it would be problematic to continue assuming that only humans can understand their own personal mortality.

## Footnotes

1 Two exceptions are Collin Allen (Allen and Hauser 1991; Allen 1999) and David Peña-Guzmán (2017).

2 For instance, one could say that perceptual access to death is a *necessary* condition for animals to acquire a concept of death, but not a sufficient one, which is why the ubiquity of death is only a *prima facie* reason for thinking some animals may understand it. To the objection that a concept of death is maladaptive, one could respond by pointing to the case of humans, who are thriving despite their knowledge and fear of death.

3 The aim of this paper does not require me to go too deeply into the debate on concept possession, since I am using the term ‘concept’ rather loosely. See footnote 5.

4 This may be an over-simplification, since recent evidence suggests that other chemicals also trigger necrophoresis in ants and other insects (Anderson et al. 2010, p. R554), but what matters is that no conceptual mediation is apparently present.

5 My ultimate aim in this paper is to provide a method to examine the amount of truth in the widespread assumption that only humans can have a concept of death. This assumption is not so much linked to our discriminatory abilities, but to the *understanding* of death that we have, which is why I follow this account of concept possession. However, my main thesis does not depend upon whether or not this account is a correct explanation of how concepts operate. For those readers who favour more demanding accounts of concept possession than the one I’m following (such as accounts that link concept possession to linguistic skills, e.g. Davidson 1982), or for readers who are uncomfortable with the use of the specific term ‘concept’ when discussing animal minds, please bear in mind that in the arguments that follow the term ‘concept’ could be easily substituted for ‘understanding,’ ‘notion,’ ‘conception,’ or any other term that could capture the sort of ability that I am referring to: an ability to *classify* individuals as dead with relative *independence of perceptual cues* and a grasp of the *intensional* features of the property of being dead. I am interested in how we could empirically determine whether animals have this particular ability. The question of whether it counts as concept possession is perhaps an interesting one, but not my research focus in this paper. In line with this, I use the terms ‘concept,’ ‘notion,’ ‘conception,’ and ‘understanding’ interchangeably throughout the paper.
